# Correspondence

**Published:** 1902-01

**Authors:** J. P. Foster

**Affiliations:** Office of State Veterinarian, Selby, S. D.


### CORRESPONDENCE.

Office of State Veterinarian,
J. P. Foster, V.S.,
Selby, S. D.

Editor Journal of Comparative Medicine, Etc.:

Dear Sir: I would like to ask if you or any of your readers can inform me if any of the best authorities on veterinary science concede that mallein is a cure for glanders? The Pasteur people, in their directions printed on a leaf which is wrapped around each bottle of their mallein and mallein solution, speak of mallein as being used "for the diagnosis *and cure* of glanders" (the italics are mine); then, in the directions for its use, it is claimed that if a glandered animal fails to react for "*two consecutive injections,*" it may be considered *cured.* I will now give you an account of a little experience I have had with this so-called *cure* (?).

About a year ago I was called to examine some horses on a farm in an adjoining county. The owner was suspicious that they were affected with glanders. I found a very bad state of affairs; out of twelve head owned on the place ten were either clinical cases or "reacters"; one of them had a complete perforation of the nasal septum, due to ulceration. Two head failed to react, and two of the "reacters" were to all appearances healthy.

I destroyed eight head and allowed the owner to keep the balance—four head—two of which, as I have mentioned, were "reacters." The barn in which they had been kept was thoroughly disinfected and given over to the cattle, and the four head of horses were removed to other out-buildings.

The two "reacters" were stabled by themselves, away from the two "non-reacters;" the harnesses were thoroughly disinfected by boiling, and the watering-troughs were properly attended to. The understanding with the owner was that he might keep the team of "reacters" until he finished his "spring's work" (providing that they did not become clinical cases in the meantime). They were to be kept entirely away from the other team, and the farm was to be under strict quarantine. After taking my departure from the farm the owner picked up one of the leaflets—which I had dropped on the barn floor—giving directions "for the diagnosis *and cure* of glanders."

He came to me a few days afterward and told me that he thought that he should have been given a chance to "cure" his horses with

ARMY MEDICAL LIBRARY
WASHINGTON, D. C. U. S. A.

mallein, and severely censured me for not informing him that mallein was a curative agent for glanders. Glanders was quite prevalent in the neighborhood, and this party told a number that they were "foolish to allow their horses to be killed when they could *cure them themselves*." Advice of this kind is not calculated to make the work of controlling an outbreak of glanders in a community any easier for the authorities. The giver of said advice sent away for a hypodermic syringe, clinical thermometer, and a supply of mallein, and began testing his team of "reacters." On April 30th he wrote me, enclosing the temperatures of a test just made, and said: "You see they do not react, and I guess they must be all right now." I wrote him that he must continue to keep his horses under strict quarantine, however, regardless of his mallein experiments. About the middle of May these horses began to discharge from the nostrils, and both were destroyed the latter part of June, with all the symptoms of glanders. I may be mistaken, but it seems to me that until it is well proven, and the veterinary profession admits that mallein *will* cure glanders, such stuff as is being spread broadcast by these people as to what mallein will do is almost criminal and on a par with the "horse book" issued by the manufacturers of spavin cures (?), which does not intimate that "farcy" is glanders or that it is incurable and dangerous to human life; and also gives a line of treatment for glanders, consisting of hyposulphite of soda, sulphate of copper, etc. I have used about 400 doses (1000 c.c.) of mallein solution put out by the Pasteur people, during the past eighteen months, and consider it all right as a *diagnostic agent;* but I think that it is about time for them to "cut out" this "cure" business.

Respectfully,

J. P. FOSTER.

---

AT Lexington, Ky., on Wednesday, December 25, 1901, Dr. John R. Hagyard, graduate of Ontario Veterinary College, class of 1875, to Miss Lula D. Potts. Such an added Christmas gift is a fitting close to the old year, and should ensure a happy and prosperous New Year.

---

THE New York County Veterinary Medical Association adopted the plan for their first meeting of the New Year of having demonstrations of a number of surgical operations. Members of the Association were selected for this part of their programme.

